# *Veterinary Research *is now a full Open Access journal

**DOI:** 10.1186/1297-9716-42-1

**Published:** 2011-01-11

**Authors:** Michel Brémont, Élodie Coulamy

**Affiliations:** 1 Veterinary Research, Unité de Virologie et Immunologie Moléculaires, INRA, Domaine de Vilvert, 78352 Jouy-en-Josas Cedex, France

## 

As previously announced in mid-2010, *Veterinary Research *became an Open Access (OA) journal, published with BioMed Central, on 1^st ^January 2011.

OA is defined as free, immediate, permanent and full-text online access for any user to peer reviewed research. Authors retain the copyright for their articles and grant anyone the right to copy, distribute or display the work provided that it is correctly cited. Some of the expected advantages of OA are increased visibility, wider audience and greater impact, accelerated publication, increased citations, and enhanced availability of online search tools.

*Veterinary Research *will continue to be supported by INRA and there is no change in the journal's title, scope of interests, editorial management, or peer-review process. The OA business model is relatively new and the cost of OA publication is a much-discussed question. The economic basis of OA is that authors, or rather their home institutions or research funders, are charged a fee to have their work published, instead of the readers being charged to access articles. The number of universities, funding agencies, and other organisations supporting OA and covering OA publication charges is growing rapidly and worldwide. A prominent example is the Harvard-initiated "Compact for Open Access Publishing Equity"[1], and more and more universities are introducing central OA funding mechanisms as the equivalents to library budgets that pay for subscriptions[2]. OA publishers like BioMed Central have membership arrangements[3] with research institutions across the globe.

For *Veterinary Research *the article processing charge for each accepted manuscript will be **500 euros in 2011**. This flat fee, which is supported by INRA and covers additional files (including movies) and data, is a highly competitive charge in the world of OA publishing.

We are convinced that publishing *Veterinary Research *as an Open Access journal will contribute to maintaining and further developing a high-quality journal in the field of the infectious diseases of animals. We look forward to working with all of you in the near future.

Best regards,

Michel Brémont

Editor-in-Chief

and

Élodie Coulamy

Editorial Assistant
